# Image and motor behavior for monitoring tumor growth in C6 glioma model

**DOI:** 10.1371/journal.pone.0201453

**Published:** 2018-07-26

**Authors:** Taylla Klei Felix Souza, Mariana Penteado Nucci, Javier Bustamante Mamani, Helio Rodrigues da Silva, Daianne Maciely Carvalho Fantacini, Lucas Eduardo Botelho de Souza, Virginia Picanço-Castro, Dimas Tadeu Covas, Edson Luis Vidoto, Alberto Tannús, Lionel Fernel Gamarra

**Affiliations:** 1 Hospital Israelita Albert Einstein, São Paulo, SP, Brazil; 2 LIM44, Faculdade de Medicina, Universidade de São Paulo, São Paulo, SP, Brazil; 3 Faculdade de Medicina de Ribeirão Preto, Universidade de São Paulo, Ribeirão Preto, SP, Brazil; 4 CIERMag-Instituto de Física de São Carlos, Universidade de São Paulo, São Paulo, Brazil; 5 Santa Casa Misericórdia de São Paulo, São Paulo, Brazil; Universidad de Castilla-La Mancha, SPAIN

## Abstract

The primary objective of this study is to monitor tumor growth by using image techniques and behavioral testing through general and specific motor activities (spontaneous movements and gait). Our sample includes male Wistar rats, 2 months old and weighing 250–300 g, that is categorized into three groups: control, sham, and experimental. The experimental group was anesthetized; the C6 cells with luciferase expression that were suspended in a culture medium were implanted into the right frontoparietal cortex of the rats. The sham group received implant only with culture medium without cells. Images and behavioral tests were evaluated at base time and at 7, 14, 21, and 28 days after induced tumor growth analysis. The tumor volume measured by magnetic resonance imaging (MRI) and quantitative bioluminescence imaging (BLI) signal showed a correlation coefficient of r = 0.96. The MRI showed that the mean tumor volume increased by approximately 10, 26, and 49 times according to a comparison of tumor volume on the seventh day with 14, 21, and 28 days, respectively. The quantification of the BLI signal was (4.12 ± 2.01) x 10^8^, (8.33 ± 3.12) x 10^8^, (28.43 ± 6.32) x 10^8^, and (63.02 ± 10.53) x 10^8^ photons/s at the seventh, fourteenth, twenty-first, and twenty-eighth day, respectively. After 14 days of tumor induction, both behavioral tests showed significant differences between tumor and sham or control groups. Our study showed a high correlation between MRI and BLI for tumor growth monitoring with complement aspects analysis in tumor volume. In addition, functional behavioral analysis displayed sensitivity to monitor tumor growth, as well as to detect early significant changes between groups, primarily in the tumor group. The results of gait analysis were more sensitive than general motor analysis.

## Introduction

Glioma is a general term used to describe a tumor that arises from the supportive cells (glial cells) of the brain that surround nerve cells and help the brain function. Currently, gliomas represent 24.7% of all primary brain tumors and 74.6% of all malignant brain tumors according to the Central Brain Tumor Registry of the United States (CBTRUS) of the American Brain Tumor Association (ABTA) [1, 2]. Gliomas vary in aggressiveness or malignancy, resulting in poor prognosis. Despite modern diagnosis and treatments, the median survival time of patients with aggressive gliomas, glioblastoma multiform (GBM), does not exceed 15 months [3].

Patients with malignant glioma often show progressive motor deficits, gait disturbances, and cognitive deterioration [4]. However, the symptoms may be nonspecific and may include headache, altered mental status, ataxia, nausea, vomiting, and weakness [5].

Among some glioma tumor models, the C6 cell line is the most similar to those reported in human brain tumors according to a review conducted with eight rat brain tumor models. In addition, the C6 glioma tumor mimics several features of human glioblastoma, including a high mitotic index, focal tumor necrosis, parenchymal invasion, and neoangiogenesis [6–8].

So far, there is a little evidence for general motor impairment of the C6 glioma tumor model to preclinical experiments [9]. Moreover, this evaluation is often seen and relevant in clinical experimentation and neurologic evaluation, such as motor impairment and gait disturbance in patients with brain glioma [10]. In other studies on glioma tumor model, complementary motor behavior evaluation of the tumor growth have been more sensitive and cost-effective functional assay for impaired behavior associated with disease progression or treatment effect [11–14]. The establishment of impaired motor signals in the C6 glioma model will facilitate the testing of new therapeutic approach for futures studies.

This study sought to monitor tumor growth by using magnetic resonance imaging (MRI) and bioluminescence imaging (BLI) techniques and behavioral testing through general and specific motor activity (spontaneous movements and gait). Behavioral testing, as a functional analysis of tumor evolution impairment, can complement the structural analysis of conventional MRI and BLI images, which are common practice in clinical studies and can prove valuable for improving the clinical relevance of translational brain tumor research. Even though behavioral testing is rarely observed in tumor model studies, this approach is very common in other brain injury models and treatment intervention studies.

In our study, the functional assay showed sensitive for early detection and longitudinally monitoring motor dysfunction, primarily in gait analysis.

## Methods

### C6 glioma cell culture

C6 glioma cells were obtained from the Rio de Janeiro Cell Bank (*Banco de Células do Rio de Janeiro* [BCRJ]). The cells were cultured in Dulbecco’s modified Eagle medium (DMEM) (Gibco, Inc. CA, USA) that was supplemented with 10% fetal bovine serum (FBS) (Gibco, Inc. CA, USA), 1% antibiotic–antimycotic solution (Gibco, Inc. CA, USA), and 1% L-glutamine (Gibco, Inc. CA, USA). The medium was changed three times a week, and the cells were grown by using 75 cm^2^ culture flasks. The cells were incubated in a humidified atmosphere with 5% CO_2_ at 37°C, until achieving the desired density of 75% confluence. Then, they were washed with phosphate-buffered saline (PBS) and detached in 3 mL of 0.04% trypsin-EDTA. Subsequently, the cells were then pelleted via centrifugation (500 g for 5 min at 21°C) and resuspended in DMEM.

Moreover, the cells were genetically engineered to generate luciferase-expressing C6 glioma cells. In brief, C6 glioma cells were transduced with VSV-G pseudotyped viruses carrying the lentiviral vector pMSCV_Luc2_T2A_Puro (provided by Dr. Deivid de Carvalho Rodrigues). The vector codifies the bioluminescent reporter luciferase-2 and the puromycin-resistance gene puromycin N-acetyl-transferase under the control of a murine stem cell virus (MSCV) promoter.

For virion production, human embryonic kidney 293FT cells grown at 80% confluence in 150 mm Petri dishes (~20 million cells/dish) were simultaneously transfected with 12 µg/dish of the vector pMSCV-Luc2-T2A-Puro along with two other helper vectors: 8 µg/dish of pCMV-dr8.91 and 4 µg/dish of pMD2.G. As previously reported, transfection was conducted with 25-kDa linear polyethylenimine (PEI, Alfa Ansar) [15]. Two days after transfection, the viral supernatant was collected and filtered by using 0.45 µm PVDF filters and concentrated by ultracentrifugation. As described in previous studies, the copy number of integrated lentiviral vector sequences was determined by quantitative real-time PCR [16].

For lentiviral transduction, virions were added to 10^6^ C6 glioma cell cultures at a multiplicity of infection of 3 (MOI = 3) in the presence of 8 µg/mL polybrene (Sigma-Aldrich). The medium was replaced after 18 h, and cells were cultured for an additional 48 h. After this period, the cells were selected for incubation with 1 µg/mL puromycin every other day for 8 days.

### Animals

We included male Wistar rats, 2 months old and weighing 250–300 g. The animals were housed at the vivarium of the Experimental Surgical Training Center (*Centro de Experimentação e Treinamento em Cirurgia* [CETEC]), and they were exposed at 21 ± 2°C with a 12 h light/dark cycle (7 a.m. to 7 p.m.). Access to food and water was *ad libitum* during the experiment. The vivarium is accredited by the Association for the Assessment and Accreditation of Laboratory Animal Care International (AAALAC International). Our study was approved by the Ethics in Animal Research Committee of the Hospital Israelita Albert Einstein (HIAE); number 2247–14.

**Fig 1:**
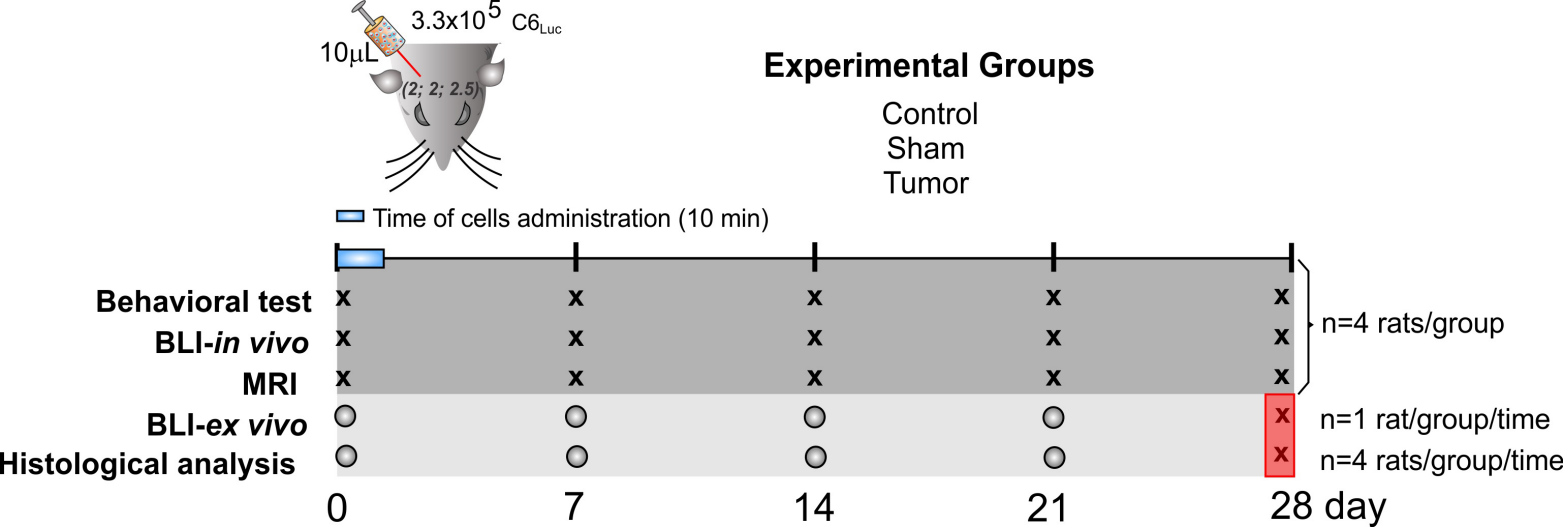
The experiment-timeline design. The picture of the rat head represents the parameters used for tumor induction: 10µL of C6Luc solution were administered during 10 min, according to the following coordinates: AP 2 mm; LL 2 mm; D 2.5 mm from bregma. The experimental groups were divided as control, sham and tumor. *In vivo* experiments comprised behavioral tests, BLI, and MRI analysis, using the same animals of each group to perform all timepoints (X) (4 rats per group, totalizing 12 rats). *Ex vivo* experiments comprised BLI and histological analysis. A new group of 48 rats was used (4 rats per each group and each time point) until the 21st day after the induction (O). 1 rat per group/time was used in the BLI analysis, and the same group of rats was used in the BLI analysis to complete the number of 4 rats per group/ time used for histological analysis. The “X” into the red square represents that the same 12 animals of the in vivo experiments were used in the last time point of the ex vivo experiments (28th day).

The experiment design comprised three groups: control, sham, and experimental/tumor, including *in vivo* and *ex vivo* experiments, as shown in the experiment-timeline design (Fig 1). The animals were randomly allocated in these groups, and they were coded and housed in individual cages before starting the experiments. The total number of animals used in this study was 60 rats. In *in vivo* procedures (Behavior test, BLI and MRI), 12 rats were used (4 rats per group) for all timepoints. For *ex vivo* procedures (histological analysis and BLI-ex vivo) 48 rats were used (4 rats per each group and each time point) until the 21st day after the induction. We used the same group of 12 animals of the *in vivo* experiments for the last time point of the ex vivo experiments (28th day), as shown in the experimental design (Fig 1).

### Stereotactic tumor induction technique

For tumor induction, animals were weighed and anesthetized with an *i*.*p*. injection of ketamine (100 mg/kg) and xylazine (20 mg/kg).

The C6 cells were implanted. The hair were removed from the top of the animal’s head. The animal was then fixed to the stereotaxic apparatus (Stoelting®, model 51700) by using in-ear and upper teeth bars. After making a skin incision on the dorsal region of the skull and removing the periosteum, a trepanning of the bone cap was done by using a dental drill. The implantation position was determined and marked on the bone according to Swanson’s Stereotaxic Atlas guidelines (1992) [17] at the following coordinates: 2.0 mm anteroposterior, 2.0 mm laterolateral, and a depth of 2.5 mm. The cells were diluted to a concentration of 10^6^ cells/30 µL. A Hamilton syringe was used to implant 10 µL of culture medium cells into the right frontal cortex. The cells were slowly injected over a 10-min period. For the control group, culture medium was injected without the cells. The syringe was kept in position for an additional 2 min before being withdrawn. To avoid drawing the injected solution back into the needle, the syringe was slowly raised until it was completely removed from the brain. The bone was then reassembled by using bone wax, and the skin was sutured using cotton thread.

### Tumor growth monitoring by MRI and bioluminescence images

The MRI and bioluminescence images were acquired at base time and at 7, 14, 21, and 28 days after tumor induction surgery for tumor growth analysis. The images were acquired after a behavior test session.

The MRI was performed on a 2T/30 cm bore superconducting magnet 85310HR model (Oxford Instruments, Abingdon, UK), which was interfaced to a Bruker Avance AVIII console (Bruker-Biospin, Inc., Billerica, MA, U.S.A) running Paravision 5.0. Software (Bruker-Biospin, Inc., Billerica, MA, U.S.A). A crossed saddle radiofrequency coil [18] was used as a head probe in rats anesthetized with an *i*.*p*. injection of ketamine (100 mg/Kg) and xylazine (20 mg/Kg).

T2-weighted images were acquired by using a rapid acquisition with relaxation enhancement sequence (RARE), with repetition time (TR)/echo time (TE) 4000/67, 1 ms, rare factor = 6, 26 slices with 1.0 mm of thickness with 0.6 mm of gap, FOV = 35 x 35 mm^2^, matrix 192 x 192, spatial resolution 182 x 182, 26 averages and a frequency of 12.5 KHz, and a total experimental time of 50 min per animal.

For morphometric analysis, the volume of the tumor was analyzed by using the Image J software (NIH, http://rsb.info.nih.gov/ij/) [19]. Total tumor volume was calculated by the sum of tumor area in each slice multiplied by the thickness and gap per slice.

In each *in vivo* BLI procedure, rats were anesthetized with isoflurane (Cristalia DCB 1565.01) by using the XGI-8 equipment (Perkin Elmer®). D-Luciferin was injected i.p. at a dose of 450 mg/kg 10 min before of the BLI acquirement in IVIS. Afterward, for *ex vivo* BLI acquiring, one animal per group of the animals that acquired the *in vivo* BLI was euthanized, and the same brain of *ex vivo* BLI was used for histological assessment. The animal/brain was then placed in the imaging chamber with 12.5 cm field of view, 2 binning factor, camera lens with aperture size F1 to maximize sensitivity, and 1-s photographic exposure time. The Images were analyzed by photons/s units by using Living Image 4.5.2 software (Xenogen).

The tumor size analyzed by MRI was correlated with the tumor sign measured by BLI at each time point, and the sensitivity of both image techniques for monitoring the tumor growth was verified.

### Behavioral testing

Animals (n = 4 per group) were placed in the behavioral test room, at least 1 h before starting the behavioral testing for habituating them, and the tests occurred between 10 a.m. and 2 p.m. The behavioral testing was realized at days 0 (baseline), 7, 14, 21, and 28 after tumor induction or sham surgery.

#### Spontaneous locomotor activity: Actimeter

Spontaneous global locomotor activity was quantified by the Infrared (IR) Actimeter LE 8825 systems (Actitrack, Panlab Harvard Apparatus, Barcelona, Spain). The apparatus comprises a two-dimensional (X-axis and Y-axis) square frame of 450 × 450 mm^2^, surrounded by transparent walls of 30 cm high, a frame support, and a control unit. Each frame counts 16 x 16 infrared beams for optimal subject detection used for evaluation of general activity, stereotyped locomotor movements, or rearings or exploration (nose-spoke detection in the hole-board option). In brief, global locomotor activity was quantified by using activity cages equipped with two horizontal infrared beams located one over the other at 4 and 8 cm above the cage floor. Each animal was placed in the center of the arena, and the spontaneous locomotor behavior was tracked for 5 min.

During the test, horizontal locomotor activity (movements or stereotype movements) was determined by breaks in movement-sensitive photobeams that were then converted into locomotor activity counts, and vertical activity was recorded as the number of rearing episodes breaking the photocell beams of the upper frame. The thresholds programmed for the upper and lower frame were 10 s and 5 s, respectively, to determine the speed of movement (slow or fast). The six parameters used for comparison between groups and sessions were slow movements (S-MOV), fast movements (F-MOV), slow stereotyped (S-STE), fast stereotyped (F-STE), slow rearing (S-REA), and fast rearing (F-REA). The data were processed using SEDACOM v2.0.

#### Gait assessment by CatWalk

The gait was analyzed by using a “CatWalk” (Noldus Information Technology, Netherlands) apparatus that comprises a 1.3-m long glass platform illuminated by fluorescent lights that are reflected downward while pressure is applied from the top. A camera is placed under the glass to record walking. The walkway was fixed to 250 mm width. The camera was positioned 70 cm below the walkway, and automatic detection settings were applied. An intensity threshold was set to 0.10, and the camera gaining was set to 9 dB. The intensity of light recorded on the CatWalk equipment represents the average brightness of all the pixels of the print on paw contact, ranging from 0 to 255 arbitrary units. A trial was regarded as successful if the animal did not have a maximum speed variation greater than 60%, with a minimum run duration of 0.5 s and a maximum run duration of 30 s, and did not stop on the runway. Any unsuccessful trial was repeated until the required number of three successful trials were achieved. Analysis was performed by using CatWalk XT 10.6. The mean of the three successful trials was used for statistical analysis.

**Fig 2:**
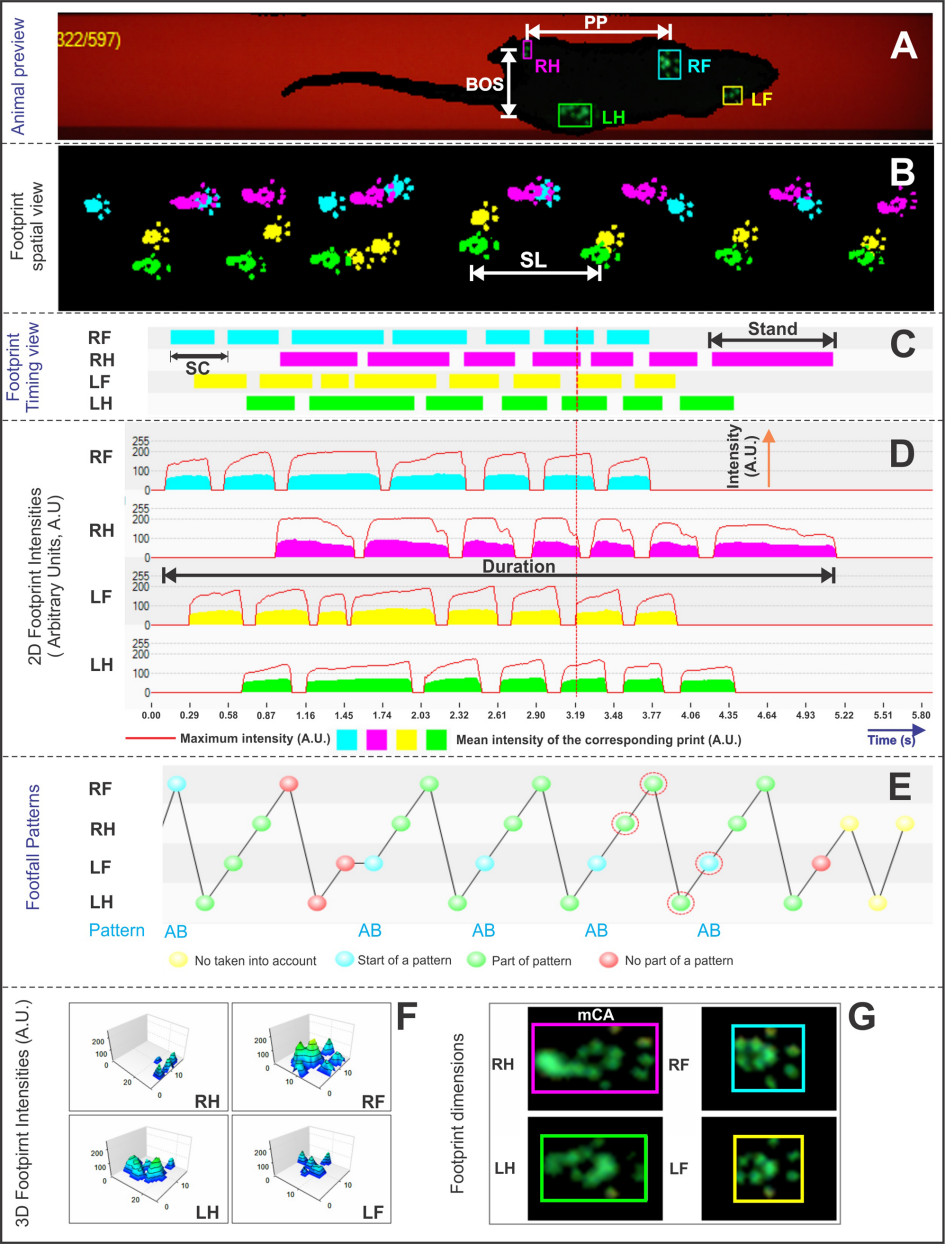
Dynamic parameters for gait analysis by CatWalk test. (A) Animal preview and automatic analysis of paws classification (RF, right forepaw in blue color; RH, right hindpaw in pink color; LF, left forepaw in yellow color; LH, left hindpaw in green color); (B) footprint spatial view (cm); (C) footprint timing view (s); (D) 2D footprint intensities measured in arbitrary units (range: 0–255 A.U.); (E) footfall patterns; (F) 3D footprint intensities (range: 0–255 A.U.); and (G) footprint dimensions of each paw (cm^2^). *Abbreviations*: PP, print positions; BOS, base of support; SL, stride length; SC, step cycle; mCA, maximum contact area; s, seconds; cm, centimeters.

During data analysis, each print on paw contact was automatically classified as right forepaw (RF), right hindpaw (RH), left forepaw (LF), and left hindpaw (LH), as shown in Fig 2A. In addition, it followed visual analysis for correct paw label (Fig 2A) and correct footfall pattern (Fig 2E), as well as faulty labels caused by tail or whiskers were eliminated. After the identification of individual footprints, we performed an automated analysis of wide-range parameters (Fig 2A–2G). Data were classified as follows:

(i) **Spatial parameters:** Print positions (PP), Fig 2A, the distance (cm) between the placement of a hindpaw and the ipsilateral frontpaw placed just before it; base of support (BOS), Fig 2A, distance between the center points of the two fore or hind paws (both represent inter-limb coordination measures); stride length (SL), Fig 2B, distance between successive placements of the same paw (dynamic gait parameters); and maximum contact area (mCA), Fig 2G, the maximum area of a paw that comes into contact with the glass plate (static gait parameters).
(ii) **Temporal parameters:** Stand, Fig 2C, the duration in seconds of contact of a paw with the glass plate; step cycle (SC), Fig 2C, the duration of stance and swing phases combined; cadence, Fig 2C and 2D, steps per second; and duration, Fig 2D, total time of entire run.

All behavioral tests were conducted by experimenters who were blinded to the treatment group. At the end of each session, animals were returned to their home cage, and the arena or walkway was wiped with 5% alcohol to avoid olfactory cues.

### Histologic assessment

The histologic assessment was realized after 1 h of baseline point of analysis in each group (control, sham, and tumor induction), as well as at 7, 14, 21, and 28 days, by using four animals per group for the analysis of each time point. Animals were fully anesthetized with an overdose of ketamine/xylazine (200 and 40 mg/kg, *i*.*p*., respectively). Moreover, they were transcardially perfused with 0.1M phosphate-buffer saline (PBS, pH 7.4), followed by 4% paraformaldehyde (PFA). Brains were removed, fixed for 24 h in 4% PFA-PB, and placed in 30% sucrose (Sigma-Aldrich, USA). After cryoprotection, coronal sections were cut into 20-µm sections on a cryostat (Leica Microsystems, Bannockburn, IL) and stained with hematoxylin and eosin (H&E) (Sigma-Aldrich, USA) for gross morphological assessment of tumors. For histological analysis of tumors, 28 serial sections of brain tissue were collected at 2.0 µm intervals processing and the images of the slides were digitized by using a ScanScope AT turbo (Aperio ®).

The protocol of this study was registered in the Protocols.io site that provided the following DOI: doi.org/10.17504/protocols.io.p4hdqt6 [PROTOCOL DOI]

### Statistical analysis

We used the statistical Package for the Social Sciences software version 24 (SPSS Inc.) [20] to analyze the behavior data. All behavior data are represented as estimated mean, and a 95% confidence interval was adopted. Motor behavior analysis experiments (gait and spontaneous movement) were analyzed by using multiple comparisons to repeat measures, and they were corrected by Bonferroni testing using the 1% level of significance. The relationship between the bioluminescence signal and intracranial tumor volume by MRI was analyzed by Pearson correlation coefficient (r), using the mean data of both images at each time point analyzed.

## Results

### Tumor growth monitoring by MRI, histology, and bioluminescence imaging

**Fig 3:**
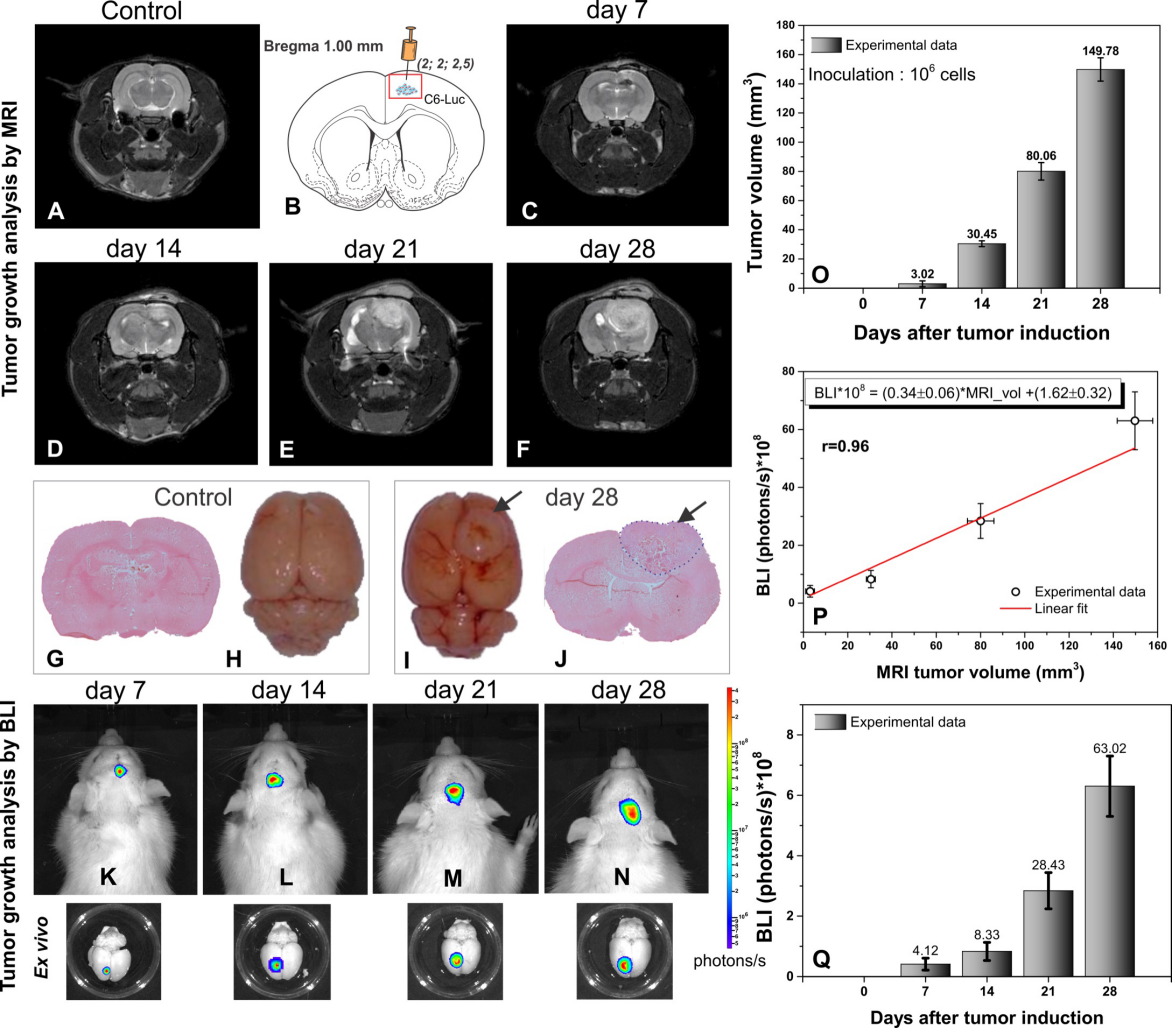
Tumor growth analysis by image. (A) Coronal plane of T2-weighted MRI images, at base; (B) C6 glioma cells injection localization according to coordinate (2.0;2;0;2.5 mm); (C–F) Coronal plane of T2-weighted MRI images at days 7, 14, 21, and 28 after tumor brain induction ().; (G–H) Microscopic and macroscopic images at base and (I–J) at the day 28 after tumor induction; (K–N) *in vivo* 2D-bioluminescence imaging at days 7, 14, 21, and days after tumor induction and corresponding brain (*ex vivo*) with bioluminescence signal after 10 min of brain extraction; (O) MRI volumetric results at each time point. (P) The mean tumor volume by MRI is plotted against the mean BLI *in vivo* signal at four points of analysis (n = 4 per group), and the Pearson correlation coefficient (r = 0.96) between both results plotted and (Q) bioluminescence tumor growth results of each time point (n = 4 per group).

*In vivo* T2-weighted MRI sequence was used to acquire the control image (Fig 3A) and after the tumor induction of C6-Luc (Fig 3B), the MRI showed an increase of the tumor over time (from day 7 to 28) as hyperintense regions (Fig 3C–3F) compared to the control group (Fig 3A). On days 21 and 28, we observed that the advanced progress of the tumor dislocated the medial line of the cortex (Fig 3E and 3F).

In addition, the histological findings (Fig 3G and 3J) and *ex vivo* macroscopic images (Fig 3H and 3I) showed the tumor evolution at control (Fig 3G and 3H) and 28 days after tumor induction (Fig 3I and 3J, tumor indicated by arrows).

The tumor growth analysis by *in vivo* and *ex vivo* BLI in Fig 3K–3N showed that *in vivo* quantifications of the BLI signals at day 7, 14, 21, and 28 was (4.12 ± 2.01) x 10^8^ photons/s, (8.33 ± 3.12) x 10^8^ photons/s, (28.43 ± 6.32) x 10^8^ photons/s, and (63.02 ± 10.53) x 10^8^ photons/s, respectively, as shown by the histogram in Fig 3Q.

The mean tumor volumes by MRI on day 7, day 14, day 21, and day 28 were 3.02 ± 1.80 mm^3^, 30.45 ± 2.10 mm^3^, 80.06 ± 6.22 mm^3^, and 149.76 ± 8.51 mm^3^, respectively. On comparing tumor volume at the seventh day with that at 14, 21, and 28 days, it was observed that the tumor volume increased by approximately 10, 26, and 49 times, respectively, as shown by the histogram in Fig 3O.

The correlation between tumor volumes as measured by MRI (Fig 3O) and the quantitative BLI signal of tumor volumes (Fig 3Q) was shown through adjusted linear determinant by BLI*10^*8*^ *= (0*.*34 ± 0*.06)*MRI_*vol*_ *+(1*.*62 ± 0*.*32)*, with a Pearson correlation coefficient r = 0.96 (Fig 3P).

### Motor behavioral changes due to tumor growth

#### Spontaneous locomotor activity analysis

**Fig 4:**
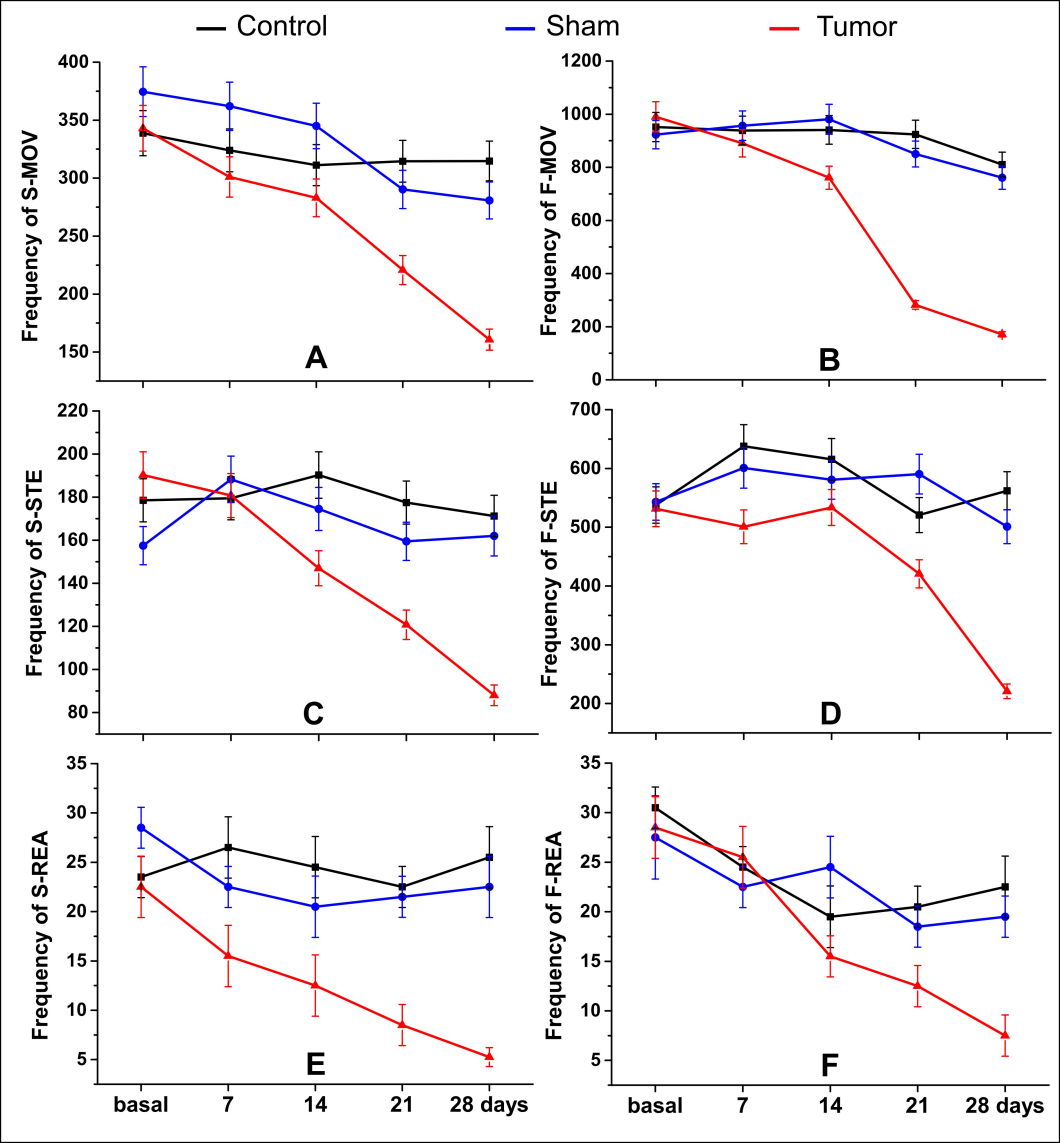
Spontaneous locomotor analysis by Actimeter. The following parameters were analyzed in slow (S) and fast (F) thresholds: (A–B) Horizontal movement (MOV), (C–D) stereotypic movements (STE), and (E–F) rearing (REA) or vertical movement. Red line: tumor group (n = 4), blue line: sham group (n = 4), and black line: control group (n = 4).

Spontaneous locomotor activity showed a significant reduction of frequency of movement (p < 0.001) mainly at later points of analysis (day 21 and 28) comparatively between the tumor group and sham and control groups for all parameters (at day 21—S-MOV: control: 314.5, sham: 290.3, and tumor: 220.8; F-MOV: control: 924.0, sham: 850.3, and tumor: 282.0; S-STE: control: 177.5, sham: 159.5, and tumor: 120.8; F-STE: control: 520.7, sham: 590.3, and tumor: 420.8; S-REA: control: 22.5, sham: 21.5, and tumor: 8.5; and F-REA: control: 20.5, sham: 18.5, and tumor: 12.5), and at day 28 after induction (S-MOV: control: 314.7, sham: 280.8, and tumor: 160.8; F-MOV: control: 810.3, sham: 760.8, and tumor: 171.3; S-STE: control: 171.3, sham: 162.0, and tumor: 88.0; F-STE: control: 562.0, sham: 501.0, and tumor: 220.8; S-REA: control: 25.5, sham: 22.5, and tumor: 5.3; and F-REA: control: 22.5, sham: 19.5, and tumor: 7.5), as shown in Table 1 and as shown in Fig 4.

**Table 1:** Estimated mean and 95% confidence interval of each group for spontaneous motor activity analysis.

Actimeter parameters (frequency)	Time (day)	Group Mean (95% CI)		
		Control (n = 4)	Sham (n = 4)	Tumor (n = 4)
**S-MOV**	0	338.8 (320.8; 357.7)	374.5 (355.6; 394.4)	343.0 (325.0; 362.0)
	7	324.0 (306.5; 342.5)	362.0 (343.5; 381.5)	301.0 (284.1; 318.9) *^§^
	14	311.2 (294.1; 329.4)	345.0 (326.9; 364.1)	283.0 (266.7; 300.3) *^§^
	21	314.5 (297.2; 332.8)	290.3 (273.7; 307.8) *	220.8 (206.4; 236.1) *^#^^§^
	28	314.7 (297.5; 333.0)	280.8 (264.5; 298.0) *	160.8 (148.6; 173.9) *^#^^§^
**F-MOV**	0	951.5 (902.1; 1003.6)	923.0 (874.3; 974.4)	990.2 (939.8; 1043.4)
	7	938.7 (889.7; 990.5)	956.8 (907.2; 1009.0)	890.3 (842.5; 940.7)
	14	940.8 (891.6; 992.6)	980.8 (930.6; 1033.7)	760.8 (716.7; 807.5) *^#^^§^
	21	924.0 (875.3; 975.4)	850.3 (803.6; 899.6)	282.0 (255.7; 311.0) *^#^^§^
	28	810.3 (764.7; 858.5) *	760.8 (716.7; 807.5) *	171.3 (151.0; 194.2) *^#^^§^
**S-STE**	0	178.5 (169.0; 188.5)	157.5 (148.6; 166.9)	190.3 (180.5; 200.5) ^§^
	7	179.5 (170.0; 189.5)	188.3 (178.5; 198.5)	180.8 (171.2; 190.8)
	14	190.2 (180.5; 200.5)	174.5 (165.2; 184.4)	147.0 (138.4; 156.1) *^#^^§^
	21	177.5 (168.1; 187.5)	159.5 (150.6; 169.0)	120.8 (113.0; 129.0) *^#^^§^
	28	171.3 (162.0; 181.0)	162.0 (153.0; 171.5)	88.0 (81.4; 95.1) *^#^^§^
**F-STE**	0	537.8 (508.0; 569.2)	543.0 (513.1; 574.6)	531.2 (501.7; 562.6)
	7	637.7 (605.3; 672.0)	600.7 (569.3; 634.0)	500.8 (472.1; 531.2) ^#^^§^
	14	615.5 (583.6; 649.1)	580.8 (549.8; 613.4)	533.5 (503.9; 564.9)
	21	520.7 (491.5; 551.8)	590.3 (559.0; 623.2)	420.8 (394.5; 448.7) *^#^^§^
	28	562.0 (531.6; 594.2)	501.0 (472.3; 531.4)	220.8 (202.0; 241.3) *^#^^§^
**S-REA**	0	23.5 (20.7; 26.6)	28.5 (25.4; 31.9)	22.5 (19.8; 25.6) ^§^
	7	26.5 (23.5; 29.8)	22.5 (19.8; 25.6)	15.5 (13.3; 18.1) ^#^^§^
	14	24.5 (21.7; 27.7)	20.5 (17.9; 23.4) *	12.5 (10.5; 14.8) *^#^^§^
	21	22.5 (19.8; 25.6)	21.5 (18.9; 24.5) *	8.5 (6.9; 10.5) *^#^^§^
	28	25.5 (22.6; 28.8)	22.5 (19.8; 25.6)	5.3 (4.0; 6.8) *^#^^§^
**F-REA**	0	30.5 (27.4; 33.9)	27.5 (24.6; 30.8)	28.5 (25.5; 31.8)
	7	24.5 (21.8; 27.6)	22.5 (19.9; 25.5)	25.5 (22.7; 28.6)
	14	19.5 (17.1; 22.3) *	24.5 (21.8; 27.6)	15.5 (13.4; 18.0) *^§^
	21	20.5 (18.0; 23.3) *	18.5 (16.1; 21.2) *	12.5 (10.6; 14.8) *^#^
	28	22.5 (19.9; 25.5)	19.5 (17.1; 22.3)	7.5 (6.1; 9.3) *^#^^§^

*Abbreviations*: S-MOV, slow movement; F-MOV, fast movement; S-STE, slow stereotypic; F-STE, fast stereotypic; S-REA, slow rearing; F-REA, fast rearing.

Multiple comparison analysis corrected by Bonferroni for time and groups.

* p < 0.001 in comparison with base time

# p < 0.001 in comparison with control group

§ p < 0.001 in comparison with sham group

In the early stages of analysis (at day 7 and 14 after induction), the significant results did not show a clear pattern of impairment. However, in the slow movements, the difference between the tumor group and sham and control groups (p < 0.001) was more evident since the day 7 of analysis: for vertical movement (S-MOV at day 7: control: 324.0, sham: 345.0, and tumor: 283.0; at day 14: control: 311.2, sham: 345.0, and tumor: 283.0) and horizontal movement (S-REA at day 7: control: 26.5, sham: 22.5, and tumor: 15.5; at day 14: control: 24.5, sham: 20.5, and tumor: 12.5), as shown in Table 1 and as shown in Fig 4A and 4E.

The time effect within the groups occurred primarily in the tumor group after the day 14 of tumor induction, as shown in Table 1 and Fig 4A–4F (red line).

No difference was observed between the control and sham groups (Table 1 and Fig 4A–4F black and blue lines).

#### Gait analysis

**Fig 5:**
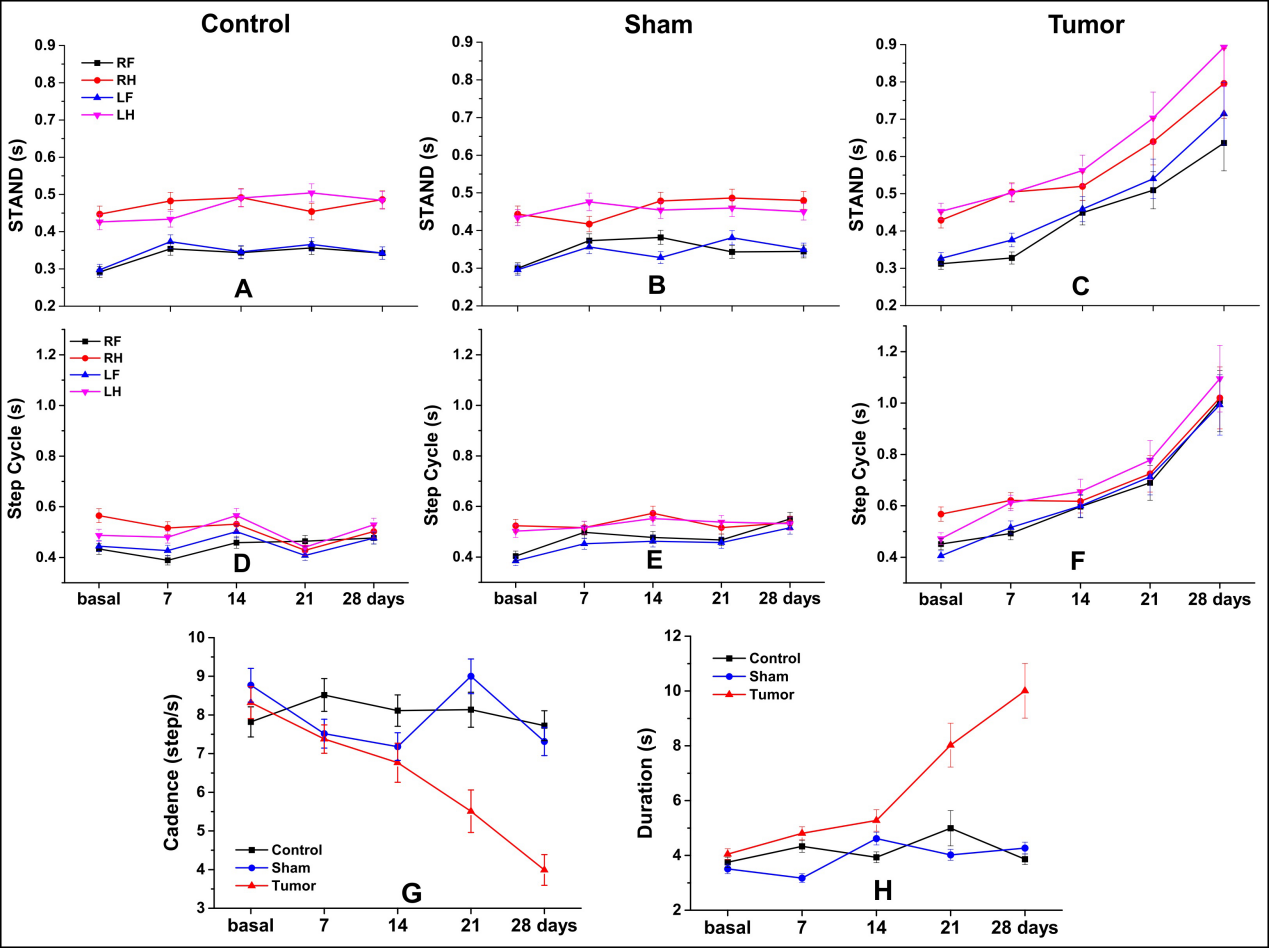
Temporal parameters of gait analysis by CatWalk test. Stand (A–C), step cycle (D–F), cadence (steps/s) (G), duration (H), for control, sham, and tumor groups (n = 4 per group) and each of the paws or side paws. Abbreviations: s, seconds; RF, right forepaw; RH, right hindpaw; LF, left forepaw; LH, left hindpaw.

**Fig 6:**
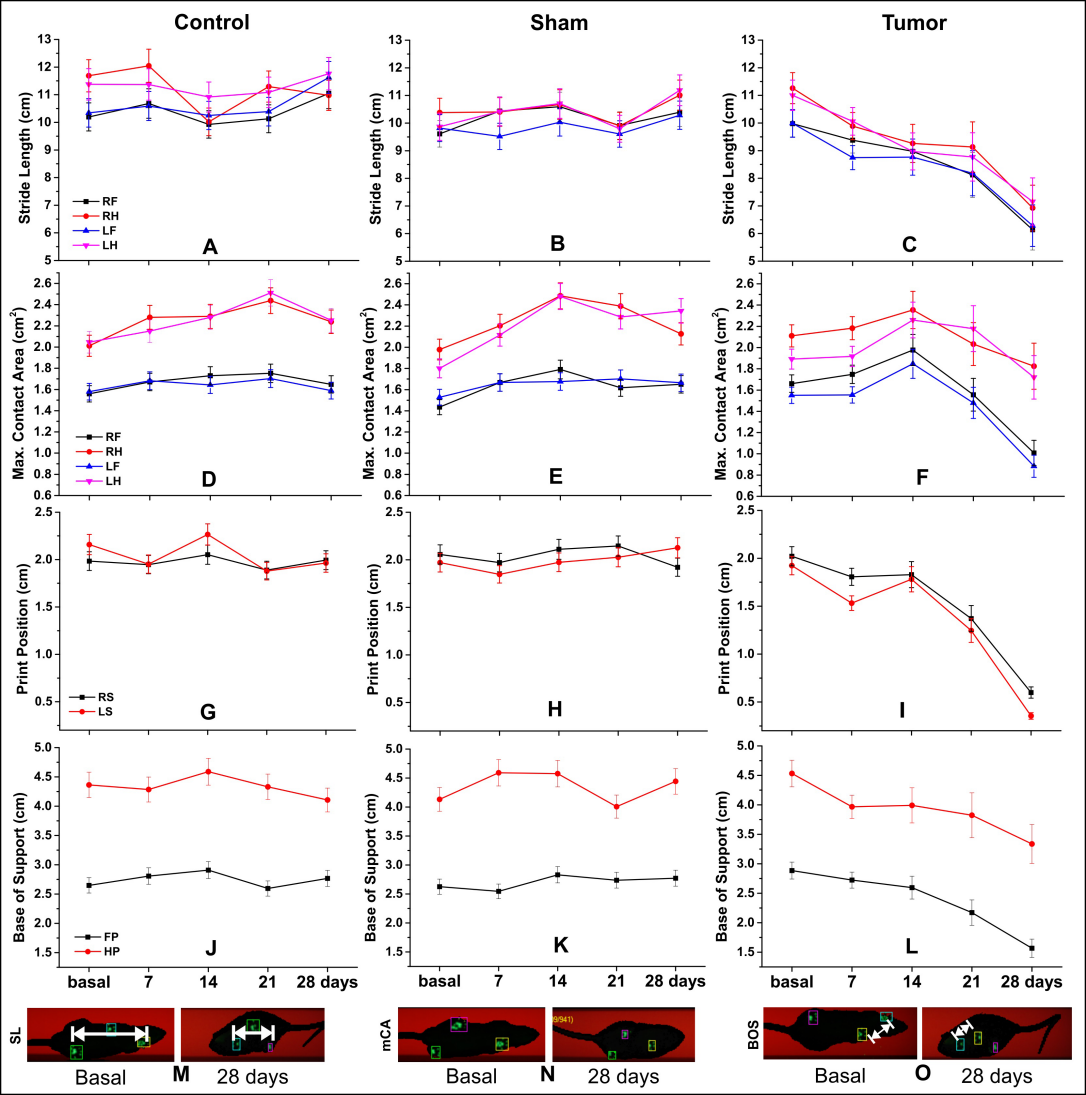
Spatial parameters of gait analysis by CatWalk test. (A–C) SL, stride length (cm); (D–F) mAC, maximum contact area (cm²); (G–I) PP, print positions (cm); (J–L) BOS, base of support (cm) for control, sham, and tumor groups (n = 4 per group) and each of the paws or side paws; (M–O) the basal and 28 days after induction spatial parameters are represented. *Abbreviations*: RF, right forepaw; RH, right hindpaw; LF, left forepaw; LH, left hindpaw; LS, left side; RS, right side; FP, forepaw; HP, hindpaw.

In gait analysis, the temporal parameters (Table 2 and Fig 5A–5H) were more sensitive to the detected significant group differences between tumor and control or sham groups than to spatial parameters (Table 3 and Fig 6A–6O). Analyses of all temporal parameters (stand, step cycle, duration, and cadence) showed relevance to specific features of gait analysis between groups and within group over time. The tumor group showed an increase of temporal parameters over day 28, with premature changes after the 7^th^ day of induction, and more consistent changes after the 14^th^ day, compared with base time; these changes are visible in Fig 5C, 5F–5H, and the significant results can be observed in Table 2.

**Table 2:** Estimated mean and 95% confidence interval of each group in some temporal parameters of gait analysis.

Catwalk parameter	Paw/Side	Time (day)	Group Mean (95% CI)		
			Control (n = 4)	Sham (n = 4)	Tumor (n = 4)
***Temporal** parameters*					
**Stand** *(s)*	**LH**	0	0.43 (0.40; 0.46)	0.43 (0.41; 0.46)	0.45 (0.43; 0.48)
		7	0.43 (0.41; 0.46)	0.47 (0.45; 0.50)	0.50 (0.48; 0.52) #
		14	0.49 (0.47; 0.52)	0.45 (0.43; 0.47)	0.56 (0.52; 0.60)* ∞
		21	0.51 (0.49; 0.52)*	0.46 (0.44; 0.48) #	0.71 (0.64; 0.78)* # ∞
		28	0.49 (0.47; 0.51)	0.45 (0.43; 0.47)	0.90 (0.79; 1.01)* # ∞
	**RH**	0	0.44 (0.43; 0.45)	0.44 (0.42; 0.47)	0.43 (0.41; 0.45)
		7	0.49 (0.47; 0.52)	0.42 (0.39; 0.44)* #	0.50 (0.48; 0.53)* ∞
		14	0.50 (0.47; 0.53)*	0.48 (0.46; 0.50)	0.52 (0.48; 0.56)*
		21	0.46 (0.44; 0.48)	0.48 (0.47; 0.50)	0.65 (0.59; 0.71)* # ∞
		28	0.48 (0.45; 0.52)	0.48 (0.46; 0.50)	0.80 (0.71; 0.90)* # ∞
	**LF**	0	0.30 (0.29; 0.31)	0.29 (0.28; 0.31)	0.33 (0.31; 0.34)
		7	0.37 (0.36; 0.38)*	0.36 (0.34; 0.38)*	0.38 (0.36; 0.39)*
		14	0.35 (0.33; 0.37)*	0.33 (0.31; 0.35)	0.46 (0.42; 0.49)* # ∞
		21	0.37 (0.35; 0.39)*	0.38 (0.36; 0.40)*	0.54 (0.49; 0.60)* # ∞
		28	0.34 (0.32; 0.35)*	0.35 (0.33; 0.37)*	0.71 (0.63; 0.79)* # ∞
	**RF**	0	0.29 (0.28; 0.30)	0.30 (0.29; 0.32)	0.31 (0.30; 0.33)
		7	0.36 (0.34; 0.37)*	0.37 (0.36; 0.39)*	0.33 (0.31; 0.34) ∞
		14	0.35 (0.33; 0.37)*	0.38 (0.36; 0.40)*	0.45 (0.42; 0.48)* # ∞
		21	0.36 (0.34; 0.37)*	0.34 (0.33; 0.36)	0.51 (0.47; 0.57)* # ∞
		28	0.34 (0.32; 0.35)*	0.34 (0.33; 0.36)*	0.64 (0.57; 0.72)* # ∞
**Step cycle** *(s)*	**LH**	0	0.48 (0.47; 0.49)	0.50 (0.48; 0.52)	0.47 (0.45; 0.50)
		7	0.48 (0.45; 0.51)	0.51 (0.49; 0.54)*	0.61 (0.58; 0.64)* # ∞
		14	0.56 (0.54; 0.59)*	0.55 (0.53; 0.57)*	0.65 (0.61; 0.70)* ∞
		21	0.45 (0.43; 0.47)	0.54 (0.52; 0.56)* #	0.78 (0.70; 0.86)* # ∞
		28	0.53 (0.51; 0.56)	0.53 (0.51; 0.56)	1.10 (0.97; 1.23)* # ∞
	**RH**	0	0.57 (0.54; 0.60)	0.53 (0.50; 0.55)	0.57 (0.54; 0.60)
		7	0.51 (0.49; 0.52)	0.51 (0.49; 0.54)	0.62 (0.59; 0.65) # ∞
		14	0.53 (0.51; 0.55)	0.58 (0.55; 0.61)* #	0.62 (0.58; 0.67)
		21	0.43 (0.41; 0.44)*	0.52 (0.49; 0.55) #	0.73 (0.66; 0.81)* # ∞
		28	0.51 (0.48; 0.54)	0.54 (0.51; 0.56)	1.02 (0.90; 1.14)* # ∞
	**LF**	0	0.44 (0.42; 0.45)	0.38 (0.36; 0.41)	0.41 (0.39; 0.43)
		7	0.43 (0.41; 0.45)	0.45 (0.43; 0.48)	0.51 (0.49; 0.54)* #
		14	0.50 (0.47; 0.52)*	0.46 (0.44; 0.48)*	0.60 (0.56; 0.65)* # ∞
		21	0.40 (0.39; 0.41)*	0.45 (0.44; 0.47)* #	0.71 (0.65; 0.79)* # ∞
		28	0.48 (0.45; 0.51)	0.51 (0.49; 0.54)*	0.99 (0.88; 1.12)* # ∞
	**RF**	0	0.44 (0.42; 0.47)	0.40 (0.38; 0.43)	0.45 (0.43; 0.47)
		7	0.39 (0.38; 0.40)	0.50 (0.48; 0.52)* #	0.49 (0.47; 0.52) #
		14	0.47 (0.44; 0.49)*	0.48 (0.45; 0.51)	0.59 (0.55; 0.64)* # ∞
		21	0.46 (0.44; 0.48)	0.47 (0.45; 0.49)*	0.70 (0.63; 0.77)* # ∞
		28	0.47 (0.45; 0.48)	0.55 (0.53; 0.58)* #	1.00 (0.89; 1.12)* # ∞
**Cadence (step/s)**		0	7.89 (7.52; 8.29)	8.77 (8.34; 9.23)	8.32 (7.92; 8.75)
		7	8.61 (8.17; 9.07)*	7.51 (7.15; 7.89) #	7.38 (7.02; 7.76)* #
		14	8.28 (7.93; 8.64)	7.19 (6.83; 7.56)* #	6.77 (6.28; 7.29)* #
		21	7.98 (7.63; 8.34)	8.93 (8.51; 9.37) #	5.51 (4.99; 6.08)* # ∞
		28	7.87 (7.56; 8.20)	7.34 (6.99; 7.72)*	3.99 (3.61; 4.41)* # ∞
**Duration** *(s)*		0	3.78 (3.61; 3.96)	3.49 (3.33; 3.66)	4.05 (3.86; 4.25) ∞
		7	4.33 (4.11; 4.56)	3.20 (3.05; 3.36) #	4.79 (4.57; 5.03)* #
		14	3.92 (3.73; 4.12)	4.65 (4.44; 4.87)* #	5.24 (4.87; 5.64)* #
		21	5.09 (4.88; 5.30)*	4.02 (3.82; 4.23)* #	7.95 (7.21; 8.77)* # ∞
		28	3.86 (3.67; 4.06)	4.29 (4.08; 4.51)*	10.10 (9.17;11.12)* # ∞

*Abbreviations*: RF, right forepaw; RH, right hindpaw; LF, left forepaw; LH, left hindpaw.

Multiple comparison analysis corrected by Bonferroni for time and groups.

* p < 0.001 in comparison to base time

# p < 0.001 in comparison to control group

∞ p < 0.001 in comparison to sham group

**Table 3:** Estimated mean and 95% confidence interval of each group in some spatial parameters of gait analysis.

Catwalk parameter	Paw/side	Time (day)	Group Mean (95% CI)		
			Control (n = 4)	Sham (n = 4)	Tumor (n = 4)
***Spatial parameters***					
**Stride length *(cm)***	**LH**	0	0.09 (0.08; 0.09)	0.10 (0.10; 0.11) #	0.09 (0.09; 0.10)
		7	0.09 (0.09; 0.09)	0.10 (0.09; 0.10)	0.10 (0.10; 0.10)
		14	0.09 (0.09; 0.10)	0.09 (0.09; 0.10)	0.11 (0.10; 0.12)* # ∞
		21	0.09 (0.09; 0.09)	0.10 (0.10; 0.11) #	0.11 (0.10; 0.13)* #
		28	0.08 (0.08; 0.09)*	0.09 (0.09; 0.09)	0.14 (0.13; 0.16)* # ∞
	**RH**	0	0.09 (0.08; 0.09)	0.10 (0.09; 0.10)	0.09 (0.08; 0.09)
		7	0.08 (0.08; 0.09)	0.10 (0.09; 0.10) #	0.10 (0.10; 0.11) #
		14	0.10 (0.09; 0.11)*	0.09 (0.09; 0.10)*	0.11 (0.10; 0.12)*
		21	0.09 (0.09; 0.09)	0.10 (0.10; 0.11)	0.11 (0.10; 0.12)
		28	0.09 (0.09; 0.09)	0.09 (0.09; 0.10)	0.15 (0.13; 0.16)* # ∞
	**LF**	0	0.10 (0.09; 0.10)	0.10 (0.10; 0.11)	0.10 (0.10; 0.11)
		7	0.09 (0.09; 0.10)	0.11 (0.10; 0.11)	0.11 (0.11; 0.12) #
		14	0.10 (0.09; 0.10)	0.10 (0.10; 0.10)*	0.11 (0.11; 0.12)
		21	0.10 (0.09; 0.10)	0.10 (0.10; 0.11)	0.12 (0.11; 0.14) #
		28	0.09 (0.08; 0.09)*	0.10 (0.09; 0.10)	0.16 (0.14; 0.18)* # ∞
	**RF**	0	0.10 (0.09; 0.10)	0.10 (0.10; 0.11)	0.10 (0.10; 0.11)
		7	0.09 (0.09; 0.10)	0.10 (0.09; 0.10)	0.11 (0.10; 0.11) #
		14	0.10 (0.10; 0.11)	0.09 (0.09; 0.10)*	0.11 (0.10; 0.12)*
		21	0.10 (0.09; 0.10)	0.10 (0.10; 0.11)*	0.12 (0.11; 0.14) # ∞
		28	0.09 (0.09; 0.10)*	0.10 (0.09; 0.10)	0.17 (0.15; 0.19)* # ∞
**Max Contact Area (cm^2^)**	**LH**	0	0.49 (0.47; 0.52)	0.56 (0.53; 0.59)	0.53 (0.51; 0.56)
		7	0.48 (0.46; 0.49)	0.47 (0.45; 0.50)*	0.52 (0.50; 0.55)
		14	0.44 (0.42; 0.46)*	0.41 (0.39; 0.43)*	0.45 (0.41; 0.48)*
		21	0.40 (0.38; 0.42)*	0.44 (0.42; 0.46)*	0.46 (0.42; 0.51)*
		28	0.45 (0.42; 0.47)*	0.43 (0.41; 0.45)*	0.59 (0.53; 0.66) # ∞
	**RH**	0	0.49 (0.47; 0.51)	0.50 (0.48; 0.53)	0.47 (0.45; 0.50)
		7	0.44 (0.43; 0.46)	0.45 (0.43; 0.48)	0.46 (0.44; 0.48)
		14	0.43 (0.41; 0.45)*	0.41 (0.39; 0.43)*	0.43 (0.40; 0.46)*
		21	0.41 (0.39; 0.44)*	0.42 (0.40; 0.44)*	0.49 (0.45; 0.54)
		28	0.45 (0.43; 0.47)*	0.47 (0.45; 0.50)	0.56 (0.49; 0.63)
	**LF**	0	0.63 (0.60; 0.67)	0.66 (0.63; 0.69)	0.65 (0.62; 0.68)
		7	0.61 (0.58; 0.63)	0.60 (0.58; 0.63)*	0.64 (0.61; 0.68)
		14	0.60 (0.57; 0.63)	0.60 (0.57; 0.63)	0.54 (0.51; 0.59)*
		21	0.59 (0.57; 0.62)*	0.59 (0.56; 0.62)	0.68 (0.62; 0.75)
		28	0.64 (0.60; 0.67)	0.61 (0.58; 0.63)	1.16 (1.04; 1.30)* # ∞
	**RF**	0	0.63 (0.60; 0.67)	0.70 (0.67; 0.73)	0.60 (0.57; 0.63) ∞
		7	0.60 (0.57; 0.63)	0.60 (0.57; 0.63)*	0.57 (0.55; 0.60)
		14	0.58 (0.55; 0.62)	0.56 (0.54; 0.59)*	0.51 (0.47; 0.55)*
		21	0.57 (0.54; 0.61)*	0.62 (0.59; 0.65)	0.66 (0.60; 0.72)
		28	0.62 (0.60; 0.64)	0.61 (0.58; 0.64)*	1.00 (0.89; 1.12)* # ∞
**Print Position *(cm)***	**LS**	0	2.15 (2.06; 2.25)	1.96 (1.86; 2.05)	1.91 (1.83; 2.01)
		7	1.94 (1.84; 2.05)	1.85 (1.76; 1.95)	1.53 (1.46; 1.60)* # ∞
		14	2.24 (2.14; 2.34)	1.97 (1.88; 2.06) #	1.78 (1.65; 1.92) #
		21	1.92 (1.84; 2.00)*	2.04 (1.95; 2.14)	1.26 (1.14; 1.39)* # ∞
		28	1.92 (1.85; 2.00)	2.14 (2.04; 2.25) #	0.35 (0.32; 0.39)* # ∞
	**RS**	0	1.98 (1.89; 2.07)	2.06 (1.97; 2.16)	2.02 (1.93; 2.12)
		7	1.95 (1.85; 2.06)	1.97 (1.88; 2.07)	1.80 (1.71; 1.89)
		14	2.06 (1.94; 2.19)*	2.10 (2.00; 2.20)	1.82 (1.69; 1.97)
		21	1.85 (1.77; 1.94)	2.15 (2.04; 2.26) #	1.37 (1.24; 1.51)* # ∞
		28	2.01 (1.91; 2.10)	1.94 (1.84; 2.03)	0.60 (0.54; 0.67)* # ∞
**Base of Support *(cm)***	**FP**	0	4.36 (4.13; 4.59)	4.14 (3.93; 4.35)	4.54 (4.33; 4.77)
		7	4.25 (4.02; 4.49)	4.57 (4.36; 4.80)	3.99 (3.80; 4.18)*
		14	4.58 (4.36; 4.81)	4.58 (4.35; 4.82)	4.01 (3.72; 4.31)
		21	4.34 (4.10; 4.60)*	3.99 (3.80; 4.18)*	3.84 (3.49; 4.23)* ∞
		28	4.14 (3.95; 4.34)	4.48 (4.27; 4.70)	3.31 (3.00; 3.65)* # ∞
	**HP**	0	2.65 (2.50; 2.80)	2.63 (2.50; 2.77)	2.88 (2.75; 3.02)
		7	2.78 (2.65; 2.91)	2.54 (2.42; 2.67)	2.73 (2.59; 2.87)* ∞
		14	2.85 (2.73; 2.99)	2.82 (2.69; 2.95)*	2.60 (2.42; 2.80)*
		21	2.60 (2.45; 2.75)	2.73 (2.61; 2.87)	2.18 (1.98; 2.41)*
		28	2.71 (2.61; 2.82)	2.76 (2.63; 2.91)	1.56 (1.42; 1.72)* # ∞

*Abbreviations*: RF, right forepaw; RH, right hindpaw; LF, left forepaw; LH, left hindpaw; LS, left side; RS, right side; FP, forepaw; HP, hindpaw.

Multiple comparison analysis corrected by Bonferroni for time and groups.

* p < 0.001 in comparison to base time

# p < 0.001 in comparison to control group

∞ p < 0.001 in comparison to sham group

We observed significant differences between the sham and control groups at some time points, mainly in step cycle, cadence, and duration parameters (Fig 5 and Table 2). Nevertheless, these significant differences in cadence and duration parameters were observed at the middle time points (day 7, 14 and 21; *p* < 0.001), but disappeared when compared to the day 28 (at day 28, mean cadence: sham: 7.34 step/s and control 7.87 step/s, *p =* 0.033; mean duration: sham = 4.29 s and control = 3.86 s, *p =* 0.004), Fig 5G and 5H. On the contrary, the differences between tumor and sham or control group increased over all time points for the same parameters, including the day 28 (at day 28 of tumor group, mean cadence = 3.99 step/s and duration = 10.10 s; *p* < 0.001 in comparison with control or sham group) (Table 2), with continuous significant changes.

As for spatial parameters (maximum contact area, stride length, print position, and base of support), the values decreased over 28 days as compared to base time, as shown in Table 3. Few spatial parameters had significant results for groups and time differences compared to temporal parameters (Tables 2 and 3).

We observed a trend of the lateralization impairment domain for some spatial parameters such as print position and maximum contact area (Table 3); whereas, this did not occur in temporal parameters (Table 2), however, neither interfered in the footfall patterns. The tumor group showed more significant changes over time than the other groups (Table 3 and Fig 6C, 6F, 6I and 6L). In addition, the sham group showed small difference than the control group (Table 3 and Fig 6B and 6H).

The stride length spatial parameter showed a homogeneous distribution of impairment, according to paw analysis. The significant results were more constant on the twenty-eighth day (tumor group in comparison to sham or control group, p < 0.001) (Table 3 and Fig 6A–6C and 6M). Fig 6M represents the significant changes in the tumor group stride length, comparing the base time image with that at day 28 after tumor induction image, as in other parameters, maximum contact area in Fig 6N, and base of support in Fig 6O.

In this study, none of the animals died during the experiment or during the follow-up period of 28 days. However, some animals were sacrificed for histological evaluation.

## Discussion

The C6 glioma tumor model with luciferase expression showed significant evolution for structural changes detected in a first measure (day 7 after implantation) by MRI and bioluminescence analysis. Moreover, it also displayed significant functional changes in general and specific motor behavior. In addition, the CatWalk test showed important aspects of temporal and spatial changes of gait associated with tumor evolution. This is a new approach in C6 glioma model study, but often explored in clinical studies of glioblastoma multiforme in which the gait instability is a common motor symptom caused by the tumor progression [10, 21–23].

The brain tumor model with the C6 cell line has been widely used in experimental neuro-oncology to evaluate the therapeutic efficacy of a variety of modalities, including studies on tumor growth, invasion, migration, and neovascularization [6–8; 24, 25]. In addition, the C6 cell line is the most similar model to those reported in human brain tumors [7]. These cells share several general histopathological and specific tumor markers with human GBM, and the tumor shows regions of focal invasion into brain tissue, which is similar to the diffuse infiltrating pattern seen in GBM [26]. Relevant aspects support our choice for the C6 glioma tumor model in this study, which can be observed in imaging results.

The MRI tumor detection and tumor growth monitoring using volumetric analysis is a useful method in preclinical and clinical studies and provides information on tumor size, location, and its relationship with adjacent structures [27]. In a preclinical study, the earliest tumor image in *in vivo* detection occurred at day 5 after tumor cell injection [28]. In this study, we observed tumor mass after 7 days of tumor injection, and tumor evolution after 21 days of injection with adjacent structure dislocation (Fig 3).

The bioluminescence analysis association can improve the spread monitoring of C6 cell luciferase implanted through a reaction of the living cell luciferase with luciferine, eliminating the influence of inflammatory tissues, necrotic tumor, or calcified tissues implicit in MRI volumetrics, a fact that can cause error in the real tumor measurements [29]. Further, the BLI is more sensitive than MRI to detect growing tumors early after implantation [30]. We observed through the first time point image measurements (day 7) that the intensity of the tumor sign area by BLI was more expressive than the tumor volume in MRI. This is relevant for therapeutic intervention studies because of the efficient measure responses in tumor regression. The *ex vivo* BLI image of this study confirmed the signal from the tumor detected in the *in vivo* BLI image (Fig 3K–3N).

Both image techniques had good correlation when compared with BLI signal and MRI tumor volume at each time point measured for tumor growth monitoring. Studies about glioma tumor model reported this correlation with excellent results [30–32]. However, this correlation can change at later stages of tumor growth. The MRI measurements showed a constantly increasing volume, whereas the BLI signal tended to plateau [30]. This pattern was not observed in our results. Both image techniques had continuous increasing values until the last measure, 28 days after tumor induction.

Brain tumor growth analysis through behavior assessment is a method that is less often used in preclinical studies with the brain tumor model [11–14, 33–35], mainly in C6 tumor glioma model [9]. However, this analysis is common with other brain injury models in preclinical studies [36–38] and in brain tumor clinical studies, evaluating some impairments, such as sensory and motor dysfunction, weakness [39, 40], and gait disturbance [40].

The general motor activity in behavior assessment is often evaluated through spontaneous locomotor activity, exploratory behavior, and coordination deficits such as open-field, cylinder, grip strength, grid walk, and rotarod tests [11–14, 33–35], or sensorimotor integration such as forelimb placing test [41]. Moreover, they represent excellent tools for the early detection and longitudinal mapping of neuronal dysfunction [42]. In addition, it shows an excellent cost-effective method for assessing disease progression or new therapeutic compounds in the preclinical model of brain tumors [43].

The spontaneous locomotor activity in the open field revealed that the tumor group showed a significant decrease of the general, stereotypic, and rearing frequency over 28 days after induction when compared with control and/or sham groups. As shown in Table 1 and shown in Fig 4, these significant differences were constant in all Actimeter parameters analyzed at days 21 and 28 after tumor induction. These behavioral changes can be associated with the glioma mass growth into the sensoriomotor cortex [44], and severe behavioral impairment affecting all motor function at day 22 with an important extension of the tumor mass [42].

During the initial phases of tumor growth (at 9 days after tumor inoculation), the muscular weakness was more sensitive of functional deficit than motor coordination that engages all the four limbs [42]: This early weakness can explain the significant change (at day 7 after tumor induction) in tumor group as compared to the control group in terms of slow movements of general motor assessment and bilateral impairment in temporal and spatial gait parameters, without the domain of laterality.

After 14 days of tumor induction, the tumor growth for the subcortical area and the behavior differences between the tumor group and control or sham groups became more evident and clearer to see in Figs 4 and 5. In the same period, a deterioration of motor performance (at 12–16 days after induction) [42] and a significant reduction of total time of the rotarod test [45] were reported.

Significant results of behavioral asymmetry were reported in the late tumor stage (at days 27 and 29 after induction) [44]. At days 21 and 28 after induction, we observed that the tumor growth compressed the contralateral brain tissue, and the bilateral behavior changes were more evident than a slight trend of unilateral spatial changes of gait assessment owing to bilateral hemisphere involvement. Severe behavioral impairment affecting all motor function was reported at day 22 with an important extension of the tumor mass [42].

Both motor activities showed significant results, but gait analysis was a more sensitive approach than spontaneous movement analysis. This was an unusual specific motor assessment for the glioma model: it showed significant changes at early time points of tumor growth and between groups, and also between sham and control groups. This difference between sham and control groups is typically explained by the effect of lesion induced by the surgical procedure in the early stage [42] and was more evidenced in temporal parameters than in spatial parameters. The spatial parameters detected differences primarily between the tumor group and the control or sham groups, and also a trend of impairment lateralization in the early stage, involving bilateral impairment in the late stage with the tumor expansion.

The structural and functional monitoring of tumor growth in a rat glioma model is essential to investigate new therapy methods [9, 11, 13, 43], although this association is not a common approach in C6 glioma model [9]. In our study, the images techniques showed a good degree of correlation with regard to tumor growth evaluation, and the general and specific motor behavioral tests had high sensitivity to detect early changes of tumor evolution. This early motor change is not common in behavior evaluation [11–13]. Moreover, the standardization of the motor signals in the C6 glioma model will facilitate the testing of new therapeutic approaches in future studies using a low cost and effective method to monitor the tumor growth.
